# The Role of Diet and Oral Supplementation for the Management of Diabetic Retinopathy and Diabetic Macular Edema: A Narrative Review

**DOI:** 10.1155/bmri/6654976

**Published:** 2025-02-24

**Authors:** Angela D'Angelo, Filippo Lixi, Livio Vitiello, Vincenzo Gagliardi, Alfonso Pellegrino, Giuseppe Giannaccare

**Affiliations:** ^1^Department of Clinical Sciences and Community Health–Department of Excellence 2023–2027, University of Milan, Milan, Italy; ^2^Department of Surgical Sciences, Eye Clinic, University of Cagliari, Cagliari, Italy; ^3^Department of Head and Neck, Eye Unit, “Luigi Curto” Hospital-Azienda Sanitaria Locale Salerno, Polla, Italy

## Abstract

Globally, diabetic retinopathy (DR) and diabetic macular edema (DME) are the leading causes of visual loss in working people. Current treatment approaches mostly target proliferative DR and DME, such as intravitreal injections of antivascular endothelial growth factor agents and laser photocoagulation. Before DR progresses into the more severe, sight-threatening proliferative stage, patients with early stages of the disease must get early and appropriate care. It has been suggested that nutraceuticals, which are natural functional foods with minimal adverse effects, may help diabetic patients with DR and DME. Several in vitro and in vivo studies were carried out over the last years, showing the potential benefits of several nutraceuticals in DR due to their neuroprotective, vasoprotective, anti-inflammatory, and antioxidant properties. Although most of the research is restricted to animal models and many nutraceuticals have low bioavailability, these compounds may adjuvate and implement conventional DR therapies. The purpose of this review is (i) to summarize the complex pathophysiology underlying DR and DME and (ii) to examine the main natural-derived molecules and dietary habits that can assist conventional therapies for the clinical management of DR and DME.

## 1. Introduction

Among several age groups, including children, working people, and the elderly, macular retinal disease has become a prominent cause of considerable vision loss due to the marked increase in the prevalence of diabetes mellitus (DM), particularly Type 2 [1]. The most frequent complications resulting in vision loss among DM patients are diabetic macular edema (DME) and diabetic retinopathy (DR) [2]. According to estimates, the number of people diagnosed with DR, vision-threatening DR, and DME globally in 2020 were 103.1, 28.5, and 18.8 million, respectively [3]. This number is expected to further rise by 2030 to 129.84 million for DR, 44.82 million for vision-threatening DR, and 23.50 million for DME [3].

Recent research points out the key role of several molecular and cellular processes, including vascular endothelial dysfunction [4], a rise in vascular permeability [5] and oxidative stress [6, 7], overexpression of adhesion molecules [8], modifications of extracellular matrix metalloproteinase (MMP) system [9], and an increase in the activity of proinflammatory molecules [10, 11].

Conversely, inflammation is a generic defensive reaction that tries to offset detrimental stimuli. It interacts with vascular alterations, but the nature of this relationship is often unclear and complicated, making predictions about its outcomes challenging [12, 13]. Furthermore, ischemia plays a significant role in the molecular mechanisms that can drive both inflammation and angiogenesis, which reinforces this relationship [14].

Considering the complex pathophysiology of DR and DME, in recent years, there has been an increasing interest in oral supplementation of natural-derived compounds and food intake for the DR and DME management [15–17]. In fact, these natural molecules can enhance the beneficial effects of conventional therapies, such as intravitreal injections of antivascular endothelial growth factor (VEGF) agents, intravitreal corticosteroid implants, laser treatment, and pars plana vitrectomy [18].

The aim of this narrative review is firstly to summarize the complex pathophysiology underlying DR and DME and then to provide the largest and updated scientific literature–based analysis of the main natural-derived molecules and dietary habits that can assist conventional therapies for the clinical management of DR and DME.

## 2. Materials and Methods

**Figure 1:**
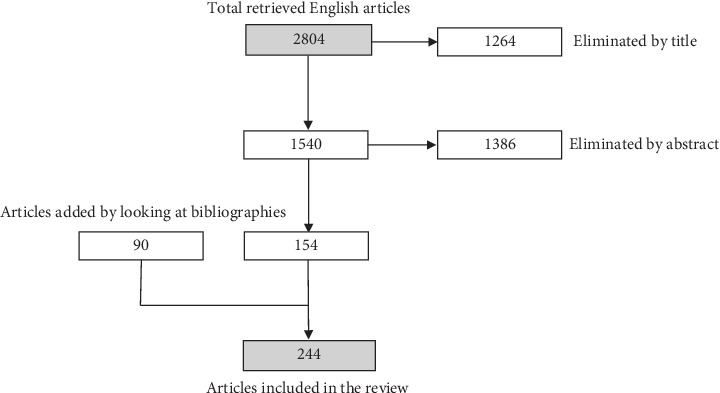
Graphical scheme of the bibliographic search.

For this narrative review, we carried out a bibliographic search on PubMed database using the following keywords: “Diabetic Retinopathy” OR “Diabetic Macular Edema” AND terms related to food intake, diet and oral supplementation (such as “carotenoids,” “lutein,” “zeaxanthin,” “astaxanthin,” “lycopene,” “crocin,” “crocetin,” “saffron,” “beta-carotene,” “polyphenols,” “flavonoids,” “resveratrol,” “curcumin,” “coenzyme Q10,” “bromelain,” “zinc,” “omega-3 fatty acids,” “Boswellia,” “vitamins,” “melatonin,” “alpha-lipoic acid,” “Ginkgo biloba,” “diet,” and “dietary habits”). The earliest publication date was January 1985, and the search ended in December 2024. Only English research articles and reviews were included. We also manually checked the reference lists of the different studies included in this review to find any additional publications that could be useful for the current paper (Figure 1).

## 3. Key Points of DR and DME Pathophysiology

Both retinal cells and vascular endothelium are significantly impacted by the dysregulation of glucose and lipid metabolism in diabetic patients. Exposure to high glucose levels has been shown to cause the death of endothelial cells and pericytes [19]; changes in the endothelium of the retinal vessels serve as chemotactic signals for the adherence of leukocytes, sustaining a chronic inflammatory cycle. In the retina, aberrant blood flow patterns present as retinal ischemia, microaneurysms, and capillary depletion all at the same time [20].

The pathophysiology of diabetic complications is often addressed within the context of cellular metabolic and signaling pathways that lead to organ dysfunction, and especially for DR/DME development, the blood–retinal barrier plays a crucial role. On the other hand, the extracellular matrix, smooth muscle cells, glia, endothelial cells, and pericytes make up the intricate multiheterocellular structures known as the neurovascular units, which represent a cooperative interaction between the neurological and vascular systems [21]. The neurovascular unit controls blood flow and retinal cell metabolism in conjunction with the blood–retinal barrier, enabling the regulated interchange of nutrients and metabolic waste products [21, 22].

The formation and progression of DR and DME are significantly influenced by cellular changes in the retinal neurovascular units, including modifications to the cytoskeleton, metabolism, chaperones, secreted proteins, signaling proteins, and transporters [23–26]. Retinal microglia, the complement system, and early innate immune system activation all contribute to retinal neurovascular units' injury by reducing synaptic protein expression and changing glial function [27, 28].

Based on available data, DR is both a neurodegenerative disease and a microvascular consequence of DM [29]. In fact, glial cells (astrocytes, Müller cells, and resident microglia) are critically positioned between the vasculature and neurons of the retina, controlling the retinal milieu, which is disrupted in the earliest stages of DR. [29] Chronic insults, such as diabetes, cause resident microglial cells to become activated, change into ameboid forms, and acquire the capacity to migrate within the retina, together with blood-derived macrophages and hyalocytes [30]. Reactive oxygen species (ROS), reactive nitrogen species, interleukin (IL)-1*β*, tumor necrosis factor (TNF)-*α*, and other proinflammatory and cytotoxic substances are produced by activated microglia [2], promoting DME development.

Hyperglycemia, together with dyslipidemia and insulin resistance, can also cause the overactivation of a number of metabolic processes in diabetic patients, such as the hexosamine pathways [31], polyol, protein kinase C activation, local renin–angiotensin system, advanced glycation end products, and epigenetic modifications [13]. In particular, the hexosamine pathway involves the phosphorylation of glucose and the subsequent transformation of glucose into fructose-6-phosphate, mediating the harmful consequences of ROS in hyperglycemia and damaging the cell endothelium [31]. Oxidative stress, hypoxia, and inflammation are all brought on by these pathways being overactivated, thus causing a breakdown of the blood–retinal barrier, raising osmotic pressure and edema [13, 32].

One of the main characteristics of proliferative DR is retinal neovascularization, which is defined by aberrant angiogenesis leading to abnormal new vessel creation, hypoxia, and vascular leakage [33]. Hypoxia and other metabolic insults to the diabetic retina have been proposed as potential causes of retinal inflammation, VEGF production, and neovascularization [34]. A dynamic layer of cells, called the vascular endothelium, communicates with other cells in the artery wall by sending and receiving intricate chemical signals, which support the vascular function and monolayer integrity [35]. Since pericytes and endothelial cells undergo rapid apoptosis in DR [36], the diabetic retinal microvasculature's equilibrium is severely upset, leading to progressive vasodegeneration [37]. When the endothelium is damaged, the inner blood–retinal barrier breaks down [38] and growth factors, cytokines, and chemokines are released locally. These molecules have a proangiogenic effect by either directly affecting endothelial cells or indirectly by causing inflammatory cells to produce angiogenic growth factors, such as VEGF. The transition to angiogenesis promotion and the development of sight-threatening DR are determined by proangiogenic cytokines, matrix proteins, growth factors, and other significant mediators in conjunction with a lack of endogenous angiostatic mediator synthesis [39].

Several changes in the retinal vasculature seen in eyes with DME may be the result of inflammatory processes [10, 11]. In fact, in the ocular fluids of DME eyes, it has been repeatedly observed that a number of cytokines, chemokines, and permeating factors, such as erythropoietin, monocyte chemoattractant protein, platelet-derived growth factor, IL-6, IL-8, and placental growth factor are elevated, with a concomitant pigment epithelium–derived factor (PEDF) decrease, which promotes neoangiogenesis.

VEGF, which is released when the retina is damaged and in presence of retinal hypoxia, can lead to neoangiogenesis but can also result in increased vascular permeability [40, 41]. In patients with DME, the levels of VEGF-A and placental growth factor are raised in the retina and vitreous, and their overexpression is linked to an increase in vascular permeability [40].

However, higher levels of inflammatory molecules, such as IL-6, IL-8, TNF receptor 1 and 2, and MMP-9, have also been reported in DME eyes that do not react well to anti-VEGF therapy [42]. TNF-*α*, IL-1*β*, and IL-6 are examples of inflammatory cytokines that are overexpressed and that encourage the overexpression of intracellular adhesion molecules, which draw in monocytes and leukocytes and sustain an ongoing inflammatory response [43, 44]. All these mechanisms cause a localized decrease in blood flow velocity, which exacerbates retinal hypoxia [43, 44].

**Figure 2:**
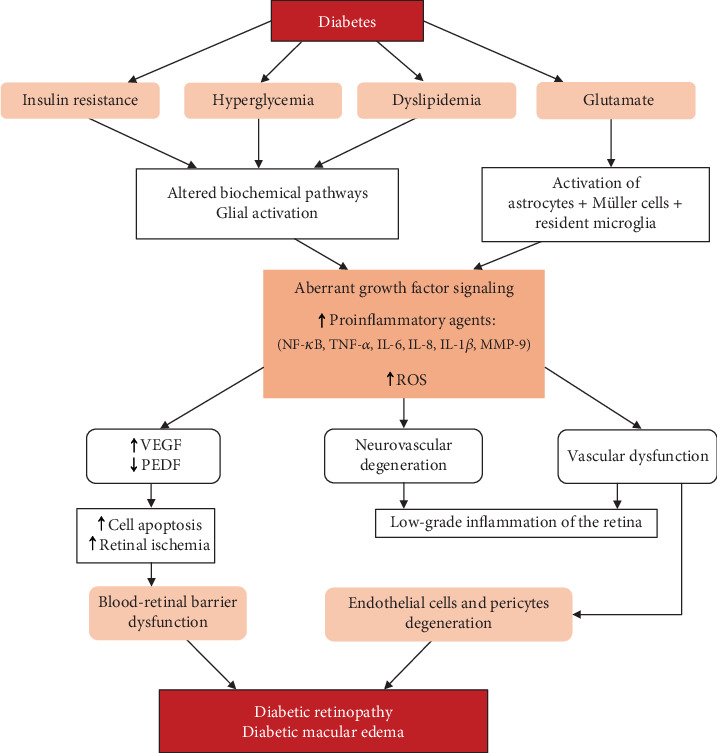
Schematic overview of the main key points of the diabetic retinopathy pathophysiology. NF-*κ*B: nuclear-kB transcription factor; TNF-*α*: tumor necrosis factor-*α*; IL-6: interleukin-6; IL-8: interleukin-8; IL-1*β*: interleukin-1*β*; MMP-9: matrix metalloproteinase-9; ROS: reactive oxidative species; VEGF: vascular endothelial growth factor; PEDF: pigment epithelium–derived factor.

The main pathways involved in the development of DR and DME are schematized in Figure 2.

## 4. Results

### 4.1. Carotenoids

#### 4.1.1. Lutein and Zeaxanthin

Lutein and zeaxanthin are plant-derived yellow–orange pigments that belong to the xanthophyll class of carotenoids, with similar structures, obtained by humans through the diet [45, 46]. High concentrations are found in green leafy vegetables like cabbage, broccoli, peas, spinach, lettuce, and egg yolks [47]. Lutein and zeaxanthin are the only dietary carotenoids that accumulate in the human eye, primarily concentrating in the macula, the central part of the retina where photoreceptor cells responsible for visual acuity and central vision are found [45, 46]. Zeaxanthin is predominantly located in the fovea, the central area of the macula, while lutein is more broadly distributed across the macula and into the peripheral retina [45, 46]. Lutein and zeaxanthin have been shown to have several beneficial properties: powerful antioxidant activity by reducing free radicals [48], a blue light filter capability by lowering phototoxic damage to photoreceptor cells [49], an anti-inflammatory activity by inhibiting the proinflammatory cytokine cascade, a neuroprotective effect by preventing the depletion of brain-derived neurotrophic factor and DNA damage [50]. Since serum and retinal levels of carotenoids have been linked with the prediction and severity of DR [51, 52], the effects of lutein and zeaxanthin supplementation on the management of DR have been extensively evaluated in *vitro*, in animal models, and in human studies. On human retinal pigment epithelial (RPE) cell line, lutein blocked high glucose–mediated elevation of intracellular ROS, reduced oxidative stress markers, and protected antioxidant enzymes [53, 54]. In in vitro models mimicking DR, antiproliferative and antiangiogenic properties have been also demonstrated [55–57]. Similar results were observed in studies on rodents, where supplementation with lutein and zeaxanthin reduced oxidative stress markers, while enhancing antioxidant enzymes [58, 59]. Additionally, these carotenoids helped maintain mitochondrial function and protect against mitochondrial stress, a key factor in the pathogenesis of DR. [59, 60] Lutein and zeaxanthin have been also demonstrated to reduce inflammation by decreasing proinflammatory mediators like nuclear-kB transcription factor (NF-*κ*B) and IL-1*β* and to mitigate changes associated with DR progression, such as increased cell permeability and neovascularization [59, 61–63]. Xanthophylls also showed neuroprotective activity, protecting retinal cells from apoptosis, preserving the structure of various retinal layers, and enhancing retinal function as measured by electroretinography [45, 58, 61, 64]. Human studies have reported significant increases in serum carotenoids and in macular levels following active oral supplementation with lutein and zeaxanthin [45, 65, 66]. Additionally, clinically significant improvements in visual performance and anatomical parameters have been observed. In one clinical trial, 90 patients were divided in three groups: one supplemented with lutein 6 mg/daily and zeaxanthin 0.5 mg/daily for 3 months, one without supplementation, and another one of healthy controls [55]. Compared to baseline, visual acuity, foveal thickness, and contrast sensitivity significantly improved in the supplemented group [55]. A retrospective study on 120 eyes of 60 patients with Type 2 DM without DR or DME revealed an increase in central foveal thickness (CFT) on OCT examination. The latter increased from 157.4 ± 13.7 to 162.8 ± 13.1*  μ*m in the right eye and increased from 157.1 ± 14 to 163.4 ± 13.2*  μ*m in the left eye, following daily supplementation with lutein (10 mg), zeaxanthin (2 mg), and meso-zeaxanthin (10 mg). In addition, retinal function within the central 13° surrounding the fovea improved after 2 years of carotenoid supplementation [67]. Conversely, in a retrospective study on 72 patients with DR divided into two groups receiving either 0.5 mg of zeaxanthin alone or 0.5 mg of zeaxanthin with an additional 6 mg of lutein daily, no differences in visual acuity, contrast sensitivity, or glare sensitivity after 4 months of oral supplementation were found [68]. Identifying effective doses of lutein and zeaxanthin remains challenging due to the limited availability of long-term clinical studies, the heterogeneity in study designs, and their variable bioavailability [69]. Nonetheless, oral supplementation with these carotenoids is considered to have a high safety profile, with a low risk of adverse effects [70].

#### 4.1.2. Astaxanthin and Lycopene

Astaxanthin and lycopene, as lutein and zeaxanthin, are xanthophylls that belong to nonprovitamin A carotenoids [71, 72]. Astaxanthin is commonly found in marine environments, where it is produced by algae and fishes and can be extracted from crustaceans [71]. Lycopene is present in various vegetables and is particularly abundant in tomatoes [73]. Both these compounds have showed various beneficial effects, including antiapoptotic, anti-inflammatory, antioxidant, and neuroprotective properties [71–73]. Astaxanthin has been demonstrated to reduce oxidative stress and inflammation, downregulating the expression of inflammatory mediators in different experimental DR models [74, 75]. In diabetic rats, astaxanthin has shown to inhibit the transcription factor NF-*κ*B and increase levels of antioxidant enzymes such as heme oxygenase 1 and peroxiredoxin [75]. Another study reported antidiabetic properties of astaxanthin treatment that, both in vitro and in vivo, decreased the activity of aldose reductase, the key enzyme in the polyol pathway which is significantly involved in DR microvascular alterations [74]. Neuroprotective effects have been also reported after astaxanthin administration in animal models [76–78], alleviating retinal tissue damage by increasing overall retinal thickness and ganglion cells by reducing apoptosis [76, 77]. Low serum lycopene levels were measured in diabetic patients, especially those with advanced DR. [79] Accordingly, its supplementation and protective activity on retina have been explored. In vitro studies demonstrated that lycopene inhibited the proliferation of human RPE cells and provided protection against cell loss caused by oxidative stress [55]. In another study, lycopene suppressed the inflammasome and cell apoptosis in retinal tissue, increased the expression of tight junction proteins, and reduced VEGF levels both on rodents and in vitro, showing antiangiogenetic action [80]. Although retinal uptake of astaxanthin and lycopene has not been clearly demonstrated and no clinical studies have specifically investigated their roles, their antioxidant, anti-inflammatory, and neuroprotective properties, comparable to those of lutein and zeaxanthin, offer a scientific basis for considering their inclusion in carotenoid-based vitamin therapy formulations in future nutraceutical trials for DR.

#### 4.1.3. Crocin and Crocetin (Saffron)

Crocin and crocetin are the main active compounds in saffron, a spice used since ancient times for its medicinal properties [81]. These compounds, classified as carotenoids, are known for their antioxidant and protective effects against ROS [81]. Research on crocin and crocetin is limited, but existing studies suggest promising results [82–84]. Crocetin has been shown to protect cells of the retinal ganglion cell-5 cell line from oxidative stress [82], while crocin treatment has been shown to reduce the production of ROS and nitric oxide in microglial cells exposed to high glucose levels [83]. An anti-inflammatory role has also been suggested for crocin, which was tested in a double-masked, placebo-controlled, randomized clinical trial, involving 60 patients with DR who had not responded to standard treatments [84]. Patients in the high-dose crocin group (15 mg per day) significantly improved their visual acuity compared to the placebo, suggesting an antioxidant and neuroprotective role of crocin in DR, mediated by an increase in blood flow, oxygen supply, mitochondrial genesis, and an improvement in inflammatory damage and oxidative stress [84].

#### 4.1.4. Other Carotenoids

Other carotenoids associated with the risk of DR include beta-carotene and provitamin A carotenoids [52, 85, 86]. One study evaluated the effects of beta-carotene (50 and 100 mg/kg) derived from palm oil mill effluent and dexamethasone (10 mg/kg) in a streptozotocin (STZ)-induced DR mouse model [85]. Treatment for 21 days improved DR-associated visual acuity responses and visual function, probably due to the antioxidant properties of beta-carotene [85].

In their study on 111 patients with Type 2 DM, Brazionis et al. observed that levels of nonprovitamin A carotenoids (such as lycopene, lutein, and zeaxanthin) were lower in those with DR compared with provitamin A carotenoids (including alpha-carotene, beta-carotene, and beta-cryptoxanthin), and a higher ratio of nonprovitamin A to provitamin A carotenoids was associated with a lower risk of DR. [52] Another cross-sectional study in 747 Chinese individuals showed that *α*-carotene levels were lower in DR patients, while *β*-carotene levels were lower in DM patients compared to a healthy, non-DM control group, suggesting a protective effect of *α*-carotene against DR and of *β*-carotene against DM [86].

These findings highlight the antioxidant role of carotenoids in the management of diabetes-related complications.

### 4.2. Polyphenol

Polyphenols, which are compounds with phenolic structural features, are known for their potent antioxidant properties [87]. These substances are commonly found in a variety of plant foods, including vegetables, fruits, coffee, tea, and wine. They can be classified into four groups: phenolic acids, flavonoids, stilbenes, and lignans [87]. Many studies have shown that polyphenols have many potential health benefits, including anticarcinogenic and neuroprotective effects [88, 89]. They can also help prevent the development of chronic diseases, like cardiovascular and neurodegenerative diseases [90].

#### 4.2.1. Flavonoids

Flavonoids are the most abundant class of polyphenols and are classified into six categories, including anthocyanins, flavonols (quercetin), and isoflavones [87]. Several in vivo and in vitro studies have demonstrated that these compounds may help to protect the blood–retinal barrier, reduce inflammation, prevent retinal thinning [91], reduce oxidative stress, and act as antioxidants in the diabetic retina [92–96]. An in vitro study using human retinal endothelial cells exposed to high levels of glucose demonstrated that blueberry anthocyanins can act as antioxidants by reducing ROS and increasing the activity of key antioxidant enzymes, including catalase and superoxide dismutase (SOD) [93]. A recent clinical trial also evaluated oral supplementation with 320 mg/day of anthocyanins for 4 weeks in patients with Type 2 DM, showing a significant reduction in proinflammatory biomarkers in the treatment group [94]. Another study has evaluated the effects of quercetin on oxidative stress, neuroinflammation, and apoptosis in the retinas of diabetic rats [95]. After 6 months of oral treatment, quercetin restored glutathione levels, increased antioxidant enzyme activity, and reduced inflammatory cytokines and the expression of NF-*κ*B and caspase-3, preventing cell death and retinal thinning [95]. The results of these studies are certainly promising, suggesting that the use of flavonoids like anthocyanins and quercetin may serve as a beneficial supplement for managing DR.

#### 4.2.2. Resveratrol

Resveratrol (3,5,4-trihydroxystilbene) is a nonflavonoid stilbene, a natural phenol produced by plants [97]. It occurs in a variety of foods such as grains, fruits, vegetables, and plant-based beverages including wine, tea, and coffee. Resveratrol exhibits a range of biological and pharmacological properties due to its strong antioxidant and anti-inflammatory effects [98]. It has been shown to be cardioprotective [99], neuroprotective [100, 101], and capable of providing antiaging benefits [98]. In vitro and in vivo studies have shown resveratrol's potential in preventing and treating diabetes [102, 103] and its use as a possible supplemental therapeutic agent for alleviating diabetic complications, including DR. [104–106] Resveratrol can reduce inflammatory mediators in diabetic neuropathy by inhibiting NF-*κ*B, thereby lowering the expression of proinflammatory factors [104]. Furthermore, in diabetic rats, resveratrol supplementation has shown significant improvements in the blood and retina, including reduced hyperglycemia, decreased oxidative stress markers, and increased SOD activity, mitigating elevated NF-*κ*B activity and apoptosis rate in the retina [105]. Additionally, resveratrol treatment has been found to alleviate diabetic effects such as increased vessel leakage, loss of pericytes, and elevated VEGF levels in the retina of diabetic mice [106]. In another study, a 2-week treatment with resveratrol, administered 6 weeks after diabetes induction in mice, significantly improved motor nerve conduction velocity, nerve blood flow, and reduced hyperalgesia [100]. Another study also demonstrated that resveratrol could reduce endoplasmic reticulum stress, which plays a pivotal role in retinal vascular degeneration [107]. A 6-month trial study on 99 patients evaluated the antioxidant efficacy of a nutraceutical formulation containing maltodextrin-encapsulated grape marc extract, rich in resveratrol, for treating DR, showing that the supplementation effectively reduced retinal swelling and oxidative stress, improving visual outcomes in DR patients [108]. In vitro, resveratrol has also demonstrated the ability to inhibit RPE cell inflammation caused by hyperglycemia [109].

#### 4.2.3. Curcumin

Curcumin, a yellow lipophilic polyphenol, is the major bioactive component of *Curcuma longa* [110]. Curcumin belongs to the group of phytochemicals and is considered a bioactive molecule with a wide range of effects, exhibiting antioxidant, anti-inflammatory, antimutagenic, and antiproliferative properties [110]. Increasing evidence suggests that curcumin may offer protection against diabetic complications [111], by delaying its progression through its anti-inflammatory and antioxidant properties [112–114]. Curcumin's therapeutic benefits in DR include reducing retinal thinning, preventing lens hardening, minimizing cell death in retinal ganglion and inner layers, reducing capillary basement membrane thickening, and safeguarding photoreceptor membranes from damage [115, 116]. Oral curcumin treatment (1 g/kg body weight) for 16 weeks in diabetic rats was demonstrated to reduce hyperglycemia, decrease levels of proinflammatory cytokines, increase antioxidant enzyme activity, and prevent retinal damage [115]. In another study on mice, curcumin treatment effectively repaired and regenerated the choroidal microvasculature in diabetic rats by improving and restoring the health of choroidal blood vessels affected by diabetes [117]. Mrudula et al. showed that in a STZ-induced DR rat model, an 8-week diet with curcumin significantly reduced VEGF expression in the retina compared to the control group [118]. A human clinical trial investigated the effects of a fixed combination of curcumin (200 mg), artemisia (80 mg), bromelain (80 mg), and black pepper (2 mg) on patients with DR and found that this oral supplementation improved central retinal thickness, visual acuity, and vessel density in people with and without DME [119]. Another clinical study [120] investigated the potential effects of the association between curcumin and *Boswellia serrata* (Retimix) on DME, since these two compounds were demonstrated to act synergistically to counteract the pathways of inflammation at multiple levels [121]. In particular, the results of this investigation suggested the protective role of this oral administration in patients with nonproliferative DR and treatment-naïve DME in maintaining baseline central macular thickness and best-corrected visual acuity over time [120]. However, despite curcumin's promising effects, it is poorly bioavailable [122], so further human studies are needed to confirm these beneficial properties.

### 4.3. Coenzyme Q10

Coenzyme Q10 (CoQ10), also known as ubiquinone, is a fat-soluble molecule that animal cells synthesize de novo [123]. CoQ10 is present in all cell membranes and primarily serves as a cofactor for mitochondrial enzymes involved in ATP production. In its reduced form, it also exhibits direct and indirect antioxidant properties [124]. In human retina, a reduction of CoQ10 levels and a consequent decrease in antioxidant ability have been observed with aging. Therefore, also considering the high metabolic activity and the high energy consumption of the retina, CoQ10 deficiency is supposed to cause retinopathies [125]. CoQ10 supplementation has been explored for its neuroprotective effects and neurotrophic effects in glaucoma [126] and retinal disorder management [127]. On human RPE cells, CoQ10 supplementation showed efficacy in preventing apoptosis and mitochondrial stress–related damage [128]. Similarly, CoQ10 eyedrops on DR-induced neurodegeneration mouse model effectively preserved the function and histology of various retinal cell types, alleviated retinal levels of MMP-9, and enhanced mitochondrial function [129]. Two randomized, double-blind, phase IIa, placebo-controlled studies confirmed the antioxidant action of CoQ10 [130, 131]. In these studies, 60 patients with nonproliferative DR without DME were randomized in three groups: one group was treated with 400 mg of CoQ10, another group was treated with a combined antioxidant therapy (10 mg of lutein, 4 mg of astaxanthin, 1 mg of zeaxanthin, 180 mg of vitamin C, 30 mg of vitamin E, 20 mg of zinc, and 1 mg of copper), and the third group was administered with placebo. A significant decrease in lipoperoxidation products, nitrites/nitrates, and total antioxidant capacity in patients managed for 6 months with CoQ10 and combined antioxidant therapy was observed [130]. Likewise, the same research group reported an increased cellular membrane fluidity of the erythrocytes, an increased fluidity of the submitochondrial particles in platelets, and a decreased ATP hydrolysis activity in the groups managed with CoQ10 and antioxidants [131]. Additionally, another randomized controlled trial further demonstrated the antioxidant effects of CoQ10 [132]. Sixty-eight patients were divided in a treatment and an untreated control group. In the 34 patients who received an antioxidant supplementation containing 50 mg Pycnogenol, 30 mg vitamin E, and 20 mg CoQ10, a significant reduction in ROS levels, from 2.64 ± 0.99 to 1.96 ± 0.35 mmol/L H_2_O_2_, was observed after 6 months. Although there were no significant changes in visual acuity, a notable decrease in central macular thickness from 223.97 ± 73.95 to 200.50 ± 56.73*  μ*m on OCT was documented, suggesting the possible role of CoQ10 in reducing vascular leakage [132]. Further research is necessarily required to determine the efficacy of CoQ10 in protecting patients with DR and their associated eye complications.

### 4.4. Bromelain

Bromelain is a plant compound derived from pineapple (*Ananas comosus*) with anti-inflammatory and antioxidant properties [133]. Bromelain has recently attracted considerable scientific interest due to its low toxicity, high efficacy, good availability, and ease of acquisition [133]. It acts as a sulfhydryl proteolytic enzyme and has been shown to have cardioprotective, immunomodulatory, and antiedema effects, making it a valuable substance in a range of dietary supplements [134].

In a nonrandomized, prospective study, 33 participants with nonproliferative DR and focal edema were treated with an oral supplement containing bromelain (500 mg) and curcugreen (200 mg) (Retinil Forte) [135]. Participants were divided into two groups: one group received the oral supplementation, and the other one served as a control. Over 12 months, the supplement group showed significant improvements in central macular thickness and vascular perfusion in the deep capillary plexus, indicating its efficacy in enhancing retinal health in DR patients [135]. In another clinical trial on 56 patients, a supplementation containing curcumin, artemisia, bromelain, and black pepper also improved central retinal thickness (290.43 ± 48.22 vs. 325.89 ± 70.66), visual acuity (0.55 ± 0.15 vs. baseline), and blood vessel density in patients with DR. [119] The mechanisms of action of bromelain are still not well understood and, for this reason, further studies are needed to fully clarify its effects on patients affected by DR and DME.

### 4.5. Zinc

Zinc is the second most prevalent trace element in the human body and is an essential micronutrient and cofactor for many enzymes and transcription factors [136]. Zinc, found in high concentrations in ocular tissues, particularly the retina and choroid, is essential for maintaining normal eye function, and its deficiency can lead to cell and tissue damage due to increased levels of free radicals [137]. As a key component of the Cu-Zn SOD enzyme, zinc helps protect cells by neutralizing free radicals [137]. It also shields protein sulfhydryl groups [138], limits free radical production by interacting with metals, and reduces inflammation by inhibiting NF-*κ*B activation through zinc finger proteins [139]. Some studies have shown that zinc levels tend to be lower in people with diabetes [140] and its supplementation may help protect against oxidative damage in early diabetes [141]. Kheirouri et al. conducted a randomized trial with 50 DR patients to assess the effects of 30 mg of zinc daily or placebo for 3 months on VEGF, brain-derived neurotrophic factor, and nerve growth factor [142]. Although zinc supplementation did not significantly alter the growth factor levels, VEGF levels were negatively correlated with zinc levels, suggesting a potential link between zinc and DR progression [142]. In conclusion, zinc reduces lipid peroxidation and damage in diabetic retina, potentially protecting against retinal degeneration and DR by controlling glucose levels and minimizing oxidative stress [143].

### 4.6. Polyunsaturated Fatty Acid (PUFA) Omega-3

Omega-3 fatty acids are essential PUFAs, which the body is not able to produce on its own and that must be obtained from the diet (chia seeds, some varieties of fish, and fish oil) [144]. Omega-3 PUFAs have been shown to have significant antioxidant and anti-inflammatory properties, making them valuable in the prevention of retinal diseases, including DR. [145, 146] Docosahexaenoic acid (DHA) is the major omega-3 PUFA in retinal photoreceptor membranes and affects membrane fluidity, permeability, and signaling mechanisms involved in phototransduction and mitochondrial function [146]. DHA levels are sensitive to oxidative stress and decrease in the retina under such conditions [145]. Other omega-3 PUFAs include alpha-linolenic acid and eicosapentaenoic acid (EPA), which help maintain a balanced retinal lipid profile and act as anti-inflammatory agents [145].

PUFAs in the retina also include omega-6, such as arachidonic acid, which contribute to membrane structure and cellular signaling [146]. However, an imbalance in the omega-6/omega-3 ratio, with an excess of omega-6, can promote proinflammatory conditions in the retina [145]. Diabetic patients often exhibit an elevated omega-6/omega-3 ratio [147], and omega-3 supplementation may help to improve this imbalance, by reducing inflammation, acting as an antioxidant, and protecting cells from ROS [145, 146]. A study by Kowluru et al. investigated the effects of omega-3 PUFA supplementation in STZ-induced DR rat model over an 11-month period. The treatment included EPA (650 mg), DHA (500 mg), alpha-linolenic acid (750 mg), and a multivitamin formulation. Results showed that omega-3 supplementation prevented the onset of DR, preserved retinal function, and reduced inflammatory markers compared to the control group [61]. The large-scale PREDIMED study was one of the first studies to show that a diet rich in omega-3 PUFAs may benefit Type 2 DM patients, with a substudy revealing a reduced incidence of DR in those who consumed at least 500 mg/day of omega-3 PUFAs, easily achieved with two servings of oily fish a week [148]. A randomized, double-masked, placebo-controlled trial also evaluated the effect of oral omega-3 fatty acid supplementation on peripheral nerve health in Type 1 diabetes [149]. Participants were randomized to receive either 1800 mg/day of omega-3 (fish oil) or 600 mg/day of placebo (olive oil) for 180 days [149]. After 6 months, the study found that those receiving omega-3 supplementation had a significant improvement in central corneal nerve fiber length compared to the placebo group, suggesting that omega-3 may protect peripheral nerves by enhancing lipid profiles, reducing inflammation, and preventing neuroapoptosis [149]. In both preclinical and clinical studies, the use of omega-3 PUFAs for the prevention and treatment of DR has shown promising results. Additional research is required to determine the role of omega-3 PUFAs in treatment and their long-term effects in DR.

### 4.7. Vitamins

Vitamins are essential micronutrients that play a crucial role in human metabolism, helping maintain overall health and preventing or treating certain diseases [150, 151]. Supplementation with these vitamins is particularly recommended when the daily intake through diet does not meet the required levels. Many studies have investigated the effect of vitamins on the prevention and treatment of various eye diseases including glaucoma, age-related macular degeneration, and DR. [126, 152, 153] Considering DR, research has evaluated two different aspects: the first one examines how the blood concentration of specific vitamins is related to this pathological condition, while the second one focuses on the effects of dietary vitamin intake on its development.

#### 4.7.1. Vitamin A

Vitamin A, also known as retinol, is a liposoluble vitamin, and its active derivative of vitamin A, 11-cis-retinal, is a critical component of rhodopsin, which contributes to light perception, while isomers such as all-trans-retinol and 11-cis-retinol are essential nutrients for retinal photoreceptors, which are vital for visual function [154]. According to a recent study, involving 11,727 people, high levels of vitamin A in the blood were associated with a lower risk of DR, especially in men and adults under 60 [155]. Another study evaluated serum zinc and vitamin A levels in 60 patients with Type 2 DM and found that lower levels of both nutrients were associated with more severe DR. [156] However, in another study, circulating vitamin A or carotenoid levels did not show a significant association with DR, unlike vitamins C, D, and E, which were found to be lower in people with DR. [157] A randomized controlled trial involving 89 preterm infants (42 supplemented and 47 controls) investigated the effect of early high-dose intramuscular vitamin A supplementation on retinal sensitivity [158]. The supplemented group received 10,000 IU of vitamin A three times a week. Results showed a significant improvement in retinal sensitivity in the supplemented group compared to the control group, suggesting that early vitamin A supplementation may enhance retinal function in infants at risk for retinopathy of prematurity [158].

There is a lack of data from other observational studies or randomized clinical trials evaluating the effects of vitamin A supplementation on DR.

#### 4.7.2. B Vitamins

The B vitamins are a group of water-soluble vitamins, and although several observational studies have investigated their association with DR in Type 2 DM patients, the results remain unclear [159–161]. Satyanarayana et al. found lower vitamin B12 levels in patients with DR, suggesting a connection to hyperhomocysteinemia [160]. Another study showed that lower blood vitamin B1 levels in Type 2 DM patients were associated with increased severity of DR. [159] Regarding vitamin B6, in one study, researchers assessed vitamin B6 intake in Japanese patients with Type 2 DM and found that higher intake was associated with a lower risk of developing DR. [162] Similarly, in another study, vitamin B6 at doses of 50–200 mg daily was associated with a lower long-term incidence of DR. [163] Moreover, for B vitamin groups, there are clinical studies that considered the combined supplementation of multiple vitamins. A study by Smolek et al. investigated the effects of a combined vitamin B6, B9, and B12 supplementation on nonproliferative DR. Seven patients completed the 6-month trial, showing significant improvements in retinal sensitivity and a reduction in central retinal thickness [164]. In a clinical trial of the Diabetes Visual Function Supplement Study, a vitamin complex containing vitamins B1, C, D3, E, omega-3 PUFAs, curcumin, zinc, and other compounds was tested on 67 patients with Type 1 and Type 2 diabetes with no retinopathy or mild to moderate nonproliferative retinopathy [65]. After 6 months, those receiving the oral supplementation showed significantly improved visual function compared to the placebo group [65]. Overall, considering the conflicting results, the effects of the B vitamin groups in DR require further investigation.

#### 4.7.3. Vitamin C

Vitamin C or ascorbic acid is a water-soluble vitamin that is found primarily in fruits and vegetables. As a potent antioxidant, it neutralizes free radicals, chelates metals, and supports neuroprotection [165]. It also promotes endothelial health, potentially improving endothelial dysfunction [165]. A study found that the mean serum vitamin C level was significantly lower in patients with DM than in those without, and DR patients had even lower levels than those with DM without DR. [166] Similarly, two observational studies have shown that people with DR have lower vitamin C levels than those without DR and healthy controls [167, 168]. Conversely, a study by Lam et al. found no significant differences in mean serum vitamin C levels among patients with DR and those without DR [169].

#### 4.7.4. Vitamin E

Vitamin E is a fat-soluble vitamin and comprises chemical compounds including tocopherols and tocotrienols and acts as a strong antioxidant, protecting against oxidative damage from free radicals [170]. Several observational studies found that individuals with DR had significantly lower serum levels of vitamin E compared to those without DR. [167, 169, 171] In a randomized, double-masked, placebo-controlled crossover trial, 36 participants with Type 1 diabetes and 9 healthy controls were assigned to receive either 1800 IU of vitamin E per day or a placebo for 4 months, followed by a crossover period for another 4 months. The study showed that vitamin E treatment significantly improved retinal blood flow in Type 1 diabetes patients, increased serum vitamin E levels, and did not affect glycemic control [172]. In another randomized trial, 55 participants with DR were treated with either vitamin E (200 mg twice daily) or a placebo for 12 months. Vitamin E significantly reduced DME and prevented an increase in retinal microbleeds, while the placebo group showed a decline in both conditions [173]. These results suggest that vitamin E supplementation may offer additional benefits in reducing the risk of developing DR, but further research is needed.

#### 4.7.5. Vitamin D

Vitamin D is an essential micronutrient that acts as a steroid hormone and is synthesized in the skin as cholecalciferol after exposure to sunlight [174]. Vitamin D is crucial for bone health [174] and has additional roles in cardiovascular function, inflammation, and immune system regulation [175, 176]. Many studies suggest an inverse correlation between vitamin D levels and DR, although the exact cause and effect remains unclear [177–179]. Zoppini et al. found an inverse and independent association between circulating 25-hydroxyvitamin D levels and the prevalence of microvascular complications in 715 patients with Type 2 DM [180]. A meta-analysis showed that diabetic patients with vitamin D deficiency had a significantly higher risk of developing DR. [181] A cross-sectional study carried out on 517 diabetic patients showed that vitamin D deficiency doubled the prevalence of DR. [182] Furthermore, a retrospective analysis of National Health and Nutrition Examination Survey showed that patients with inadequate vitamin D and poor disease management had a higher prevalence of both mild and severe DR than those with adequate vitamin D levels [183]. It is still unclear whether low vitamin D levels contribute to the development of DR or whether DR itself lowers vitamin D levels [184]. Several studies suggest that vitamin D may help prevent DR through its anti-inflammatory and antiangiogenic effects [185]. Moreover, vitamin D may influence the progression of DR by regulating the immune system and reducing inflammatory proteins [186] and proinflammatory cytokines, such as TNF-*α* and IL-6 [187]. It also may inhibit angiogenesis, a key factor in the development of DR, as demonstrated by the ability of calcitriol supplementation to reduce angiogenesis in the choroidal vasculature in both ex vivo and in vivo studies [188]. However, there is a lack of prospective studies evaluating the effect of vitamin D supplementation on DR in humans. In a randomized clinical trial of 83 people with DME, correcting vitamin D deficiency with an oral supplementation along with intravitreal bevacizumab treatment significantly improved visual acuity and central macular thickness [189]. A 12-week trial in 48 patients with Type 2 DM showed that vitamin D supplementation after 3 months reduced biochemical markers, IL-6, fasting insulin, and homeostatic model assessment for insulin resistance levels [190]. Further trials are absolutely needed to better understand the effects of vitamin D on DR.

An overview of the potential role of vitamins in DR management is summarized in Table 1.

### 4.8. Melatonin

Melatonin is a neurohormone produced by the pineal gland [191], and its relationship with the eyes, especially retinal ganglion cells, has received much attention in ophthalmology. Melatonin has been found to inhibit the pathway that maintains the integrity of the blood–retinal barrier in experimental DR, involving phosphoinositide 3-kinase, protein kinase B, signal transducer and activator of transcription 3, and NF-*κ*B [192]. Moreover, variations in the genes encoding melatonin synthase and receptors have also been linked to the pathophysiology of Type 2 DM, according to genome-wide association studies [193, 194]. Melatonin seems to have several biological roles in the human body, including neuroprotective, anti-inflammatory, and antioxidant properties. In age-related macular degeneration and DR, it was demonstrated to protect ocular tissues from oxidative stress and inflammatory damage by scavenging free radicals, reducing the formation of inflammatory compounds, and reducing inflammation [195]. Melatonin also controls the development of fundus degenerative lesions by preventing neovascularization and vascular leakage [196]. For this reason, through its antiangiogenic and anti-inflammatory properties, clinical and laboratory research pointed out its significance in regulating vasomotor function, preserving fundus vasculature, and averting problems including fundus bleeding. In addition, the antioxidant properties of melatonin help to repair, preserve, and normalize the turnover of retinal tissue and shield retinal cells from apoptosis [197]. However, even with these encouraging results, further clinical studies on humans are needed to maximize the therapeutic use of melatonin.

### 4.9. Oleanolic Acid

Oleanolic acid, a natural pentacyclic triterpene, has gained attention for its therapeutic potential in treating inflammation, diabetes, and related organ damage [198]. Oleanolic acid has shown promising effects in reducing diabetic complications by inhibiting the formation of advanced glycation end products [199, 200]. A study showed that oleanolic acid reduced fructosamine and advanced glycation end products, protected proteins from sugar damage, and scavenged harmful substances, suggesting that it could be a powerful antiglycation agent for preventing diabetic complications such as retinopathy [199]. Another study evaluated the Zhujing Pill, a combination of crude extracts of medicinal plants, such as Goji berries and Plantadodder seeds, for the treatment of DR and showed that oleanolic acid was the main active ingredient that improved DR symptoms in animal studies [200]. However, more research is needed to understand the effects of this promising bioactive compound.

### 4.10. Alpha-Lipoic Acid (ALA)

ALA is a potent antioxidant and experimental evidence suggests its potential role as a pharmacological agent for DR. [201] A study in 32 preretinopathic diabetic patients found that 400 mg/day of ALA combined with genistein and vitamins for 2 months protected retinal cells from oxidative stress [202]. In contrast, a randomized trial with 467 patients with Type 2 DM treated with 600 mg/day of ALA or placebo for 2 years showed no effect on preventing DME [203]. Further research is necessary to determine the efficacy of ALA in DR.

### 4.11. *Ginkgo biloba*

*Ginkgo biloba* is a living fossil, as it is one of the most ancient living trees that has existed for more than 250 million years [204]. Given its antioxidant and platelet antiaggregation properties as well as other benefits, including dilated blood vessels and better abnormal blood rheology, *Ginkgo biloba* extracts have garnered increasing interest [205]. Recent research suggests that *Ginkgo* is a useful tool for halting the progression of DR and lowering its incidence, together with the decrease of oxidative stress [206, 207]. In addition, one potential mechanism for *Ginkgo biloba* extracts' anti-inflammatory benefits is their capacity to suppress platelet-activating factor expression. Under hypoxic circumstances, they may also dramatically lower the transcriptional expressions of VEGF and hypoxia inducible factor in RPE cells [208]. The anti-inflammatory impact of *Ginkgo* also seems to be mostly dependent on IL modulation [204]. For this reason, *Ginkgo biloba* extracts help patients with DR by improving the blood flow to the retinal capillaries. In diabetic individuals, proper *Ginkgo biloba* extract administration could reduce the occurrence and development of this potentially blinding condition, suggesting its potential applications in the management and prevention of DR.

### 4.12. Diet

Although diet plays a significant role in the DM development [209, 210], its role in DR is less clear due to limited research in this area [211]. Only one study has evaluated the relationship between overall diet and DR [212], while other studies have investigated intake of specific food groups [148, 213], macronutrients [214–217], micronutrients [218–221], or beverages [222, 223].

The clinical trial by Diaz-Lopez et al. involved over 3600 patients with Type 2 DM with high cardiovascular risk [212]. The participants were randomly assigned to one of three diets: a Mediterranean diet with extra virgin olive oil, a Mediterranean diet with mixed nuts, or a low-fat control diet. Only the Mediterranean diet with olive oil was significantly associated with a more than 40% reduction in the DR risk [212]. The Mediterranean diet, rich in whole grains, fruits, vegetables, plant-based proteins, fish, olive oil, and low-fat dairy, is associated with a lower risk of DM [224], and its components also provide strong protection against DR. It has been observed that a high consumption of fruit (≥ 170 g per day) was associated with a reduction in the risk of incident DR of more than 50%, due to its content of antioxidants, vitamins C and E, carotenoids, and polyphenols [213]. Eating fatty fish at least twice a week (compared with less than twice a week) was associated with a 60% reduction in the DR risk [148]. Garlic has also been studied as a dietary supplementation for effects that appear to be beneficial in DR. [225, 226] In fact, a recent review examines in vitro, in vivo, and clinical studies from 1980 to 2022, revealing that garlic has antidiabetic, antiangiogenic, and neuroprotective properties [226]. In a clinical trial, 91 diabetic participants were randomized to receive either garlic tablets (500 mg, 2 tab/day) or a placebo for 4 weeks, with the garlic group showing significant improvements in visual acuity and reduced intraocular pressure, highlighting garlic's potential as a well-tolerated complementary treatment for DME [225].

On the other hand, the results concerning the alcohol consumption are inconsistent. In fact, most studies have found no association between alcohol consumption and DR [222, 227], while other investigations have found a positive association with DR or DME [228]. Conversely, tea consumption has been associated with a reduced risk of developing Type 2 DM [229] and has a protective effect in the DR prevention [223]. As many aspects of diet remain unexplored, further research is absolutely needed to better understand the relationship between dietary habits and DR/DME.

## 5. Discussion

DR is a dangerous DM complication that can cause visual loss and worse quality of life. Mild, subclinical abnormalities in DR proceed to moderate or severe phases, which are characterized by the formation of new vessels, retinal ischemia, and consequent vision impairment or loss [230]. Nowadays, there are few strategies available for the early stage of the disease, when retinal loss is minimal or absent and a therapeutic intervention could be most effective in stopping the progression of retinal damage, avoiding a sight-threatening retinopathy. In fact, the current DR treatment is mainly based on invasive and expensive procedures (laser photocoagulation, vitrectomy, intravitreal drugs) only for the management of the advanced stages of the disease [230]. Considering this scenario, new noninvasive and less expensive treatment options, coupled with screening programs and the conventional therapies, would be very useful, also considering the pandemic rise in DM and the number of people at risk of DR-induced visual loss. For this reason, several clinical and preclinical studies that looked at the potential effects of nutraceuticals on DR and DME were carried out in the last years [231, 232]. Moreover, DM itself may change nutritional status, reflecting a patient's perspective on micronutrient deficiencies [160]. .Overall, a wealth of data has shown how promising nutraceuticals may be used to manage DR (Table 2).

**Figure 3:**
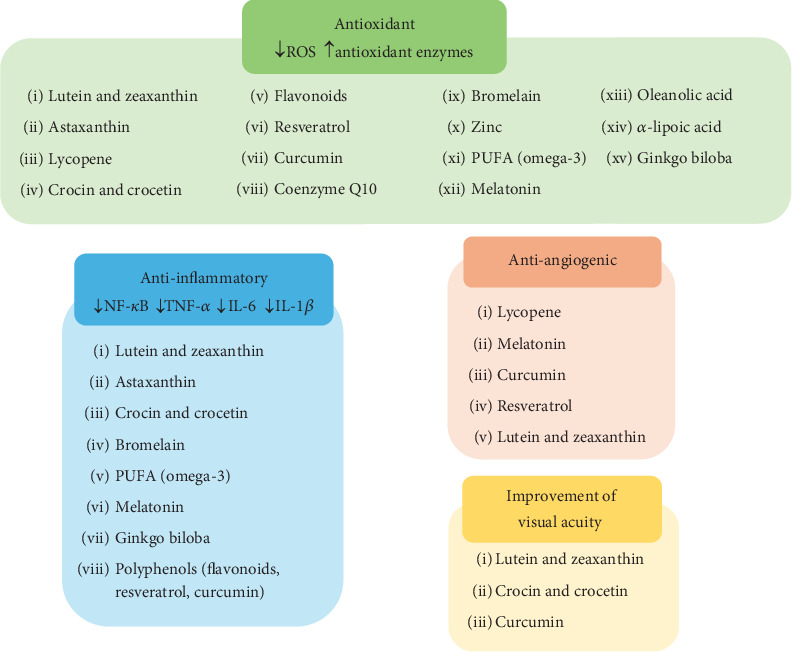
Graphical summary of the beneficial effects of the analyzed bioactive compounds in diabetic retinopathy. ROS: reactive oxygen species; PUFAs: polyunsaturated fatty acids; NF-*κ*B: nuclear-kB transcription factor; TNF-*α*: tumor necrosis factor-*α*; IL-6: interleukin-6; IL-1*β*: interleukin-1*β*.

Researchers are motivated to investigate the fundamental mechanisms of these nutraceuticals in models related to DR. Many nutraceuticals exhibit anti-inflammatory and antioxidant properties and antiangiogenic effects and may enhance visual acuity (Figure 3).

Some of them may enhance retinal vasculature and function while protecting neurons, neuroglia, and epithelial cells. In DR, retinal ganglion cells, Müller cells, and photoreceptors are damaged by neurodegeneration and apoptosis primarily driven by oxidative stress [233]. Certain flavonoids may be useful in the prevention or treatment of DR because they have anti-inflammatory, antiangiogenic, and antioxidant properties [234, 235]. Quercetin shields ganglion cells from apoptotic cell death by lowering oxidative stress, neuroinflammation, and apoptosis [95]. Additionally, resveratrol inhibits the expression of genes related to angiogenesis, inflammation, and oxidative stress in the retina [236], stopping retinal cell death, inflammatory cytokine release, and retinal thinning [237, 238]. The antioxidant and antiangiogenic properties of curcumin contrast oxidative stress and protect Müller cells, preventing the downregulation of glutamine synthase in the retina [239]. For these reasons, a continuous nutraceutical supplementation combined with a healthy diet may provide sufficient antioxidant capacity, and nutraceutical-based strategies may be effective, affordable, and long-lasting ways to slow or even stop the progression of DR in patients with DM. However, as several clinical studies have shown, some well-known molecules, such as omega-3 PUFAs, have shown lower therapeutic efficacy when used in clinical practice. Furthermore, many carotenoids and polyphenols that resemble nutraceuticals have low bioavailability, which is defined as low bioaccessibility, absorption rate, or transformation. This makes their practical application difficult. In addition, some people with DM may have impaired liver and kidney function, making it impossible for them to take higher dosages of these nutraceuticals.

In addition to their beneficial effects, possible adverse effects of these bioactive compounds should also be considered when used for DR management. All the analyzed bioactive compounds have demonstrated significant protective effects on the retina in case of DR, both in terms of lowering the disease's risk of onset and progression. When taken orally, all these molecules have a favorable safety and tolerability profile and a moderate to high bioavailability, except for curcumin, resveratrol, flavonoids, oleanolic acid, and lycopene (Table 2).

All the described compounds have shown minimal toxicity in the clinical research reported in the literature, with the exception of curcumin [122], coenzyme Q10 [240], ALA [241], melatonin [242], PUFA [243], and zinc [244], which have shown some adverse effects and drug interactions (Table 2).

Among the limitations of this review, its narrative and nonsystematic nature as well as the use of only one scientific database (PubMed) should be mentioned.

## 6. Conclusions

The multifactorial pathophysiology of DR, comprising the interplay between the detrimental effects of hyperglycemia, dyslipidemia, hypoxia, and ROS, presents numerous pathways potentially addressed by nutraceuticals. Despite their low bioavailability and efficacy compared to medications, they are nontoxic and widely available and have minimal side effects. Strong anti-inflammatory and antioxidant properties of nutraceuticals make them promising for retinoprotective prophylaxis in nonproliferative DR and probably proliferative DR, potentially improving patients' quality of life. However, further clinical studies are needed to better understand the beneficial role and validate the efficacy of nutrients and nutraceuticals in the management and prevention of DR and DME.

## Figures and Tables

**Table 1:** Overview of the potential role of vitamin supplementation on diabetic retinopathy.

**Vitamins**	**Effects**	**Serum level**	**Supplementation**
Vitamin A	—	↑ vitamin A ↓ DR risk, adults < 60 years [155] ↓ vitamin A ↑ DR severity [156]	↑ retinal function in infants at risk of retinopathy of prematurity [158]
B vitamins	—	↓ vitamin B12 in DR patients [160] ↓ vitamin B1 ↑ DR severity [159]	Vitamin B6 was associated with a lower long-term incidence of DR [163] Vitamins B6, B9, and B12 ↑ retinal sensitivity ↓ central retinal thickness in nonproliferative DR [164] Vitamins B1, C, D3, and E ↑ visual function [65]
Vitamin C	Antioxidant activity	↓ vitamin C in DR patients [166–169]	Vitamins B1, C, D3, and E ↑ visual function [65]
Vitamin E	Antioxidant activity ↑ retinal blood flow	↓ vitamin E in DR patients [167, 168, 171]	↑ retinal blood flow in Type 1 diabetes patients, not affecting glycemic control [172] ↓ diabetic macular edema and prevented retinal microhemorrhages [173]
Vitamin D	Anti-inflammatory effects Antiangiogenic effects	↓ vitamin D ↑ DR risk [181]	↑ visual acuity and central macular thickness [189] ↓ biochemical markers, interleukin-6, fasting insulin, and homeostatic model assessment for insulin resistance levels [190]

Abbreviation: DR, diabetic retinopathy.

**Table 2:** Overview of the main effects of the discussed supplement compounds for the management of diabetic retinopathy.

**Active compounds**	**Effects**	**Bioavailability**	**Characteristics**
*Carotenoids*			
Lutein and zeaxanthin	Antioxidant activity -↓ ROS -↑ antioxidant enzymes Anti-inflammatory effects -↓ proinflammatory cytokines -↓ NF-*κ*B Improvement of visual function -↑ visual acuity -↑ contrast sensitivity	Moderate	Safety profile with low risk of adverse effects

Astaxanthin	Antioxidant activity -↓ ROS -↑ antioxidant enzymes Anti-inflammatory effects -↓ NF-*κ*B	Low/moderate	Minimal toxicity

Lycopene	Antioxidant activity Antiangiogenetic action -↓ growth of RPE cells	Low/moderate	Minimal toxicity

Crocin and crocetin	Antioxidant activity -↓ ROS Anti-inflammatory effects Improvement of visual acuity	Moderate	May interfere with cardiovascular drugs

*Polyphenols*			
Flavonoids	Antioxidant activity -↓ ROS -↑ antioxidant enzymes Protection of the blood–retinal barrier Anti-inflammatory effects Prevention of retinal thinning	Low	No risk of adverse effects demonstrated May interfere with some drugs

Resveratrol	Antioxidant activity Anti-inflammatory effects -↓ NF-*κ*B -↓ TNF-*α* and IL-6	Very low	No risk of adverse effects demonstrated May interfere with certain enzymes

Curcumin	Antioxidant activity -↑ antioxidant enzymes Anti-inflammatory effects -↓ proinflammatory cytokines ↓ retinal thinning Improvement of visual acuity	Very low	Minimal toxicity, predominantly gastrointestinal upsets May interfere with antibiotics, cardiovascular and anticancer drugs

*Other compounds*			
Coenzyme Q10	Antioxidant activity -↓ ROS	Moderate/high	May interfere with warfarin, antihypertensive and chemotropic drugs

Bromelain	Antioxidant activity Anti-inflammatory effects	High	Low toxicity, high efficacy, good availability, and ease of acquisition

Zinc	Antioxidant activity -↓ ROS -↓ lipid peroxidation -↓ NF-*κ*B	High	May cause copper deficiency anemia Potential negative effects on retinal cells

PUFA (omega-3)	Antioxidant activity -↓ ROS Anti-inflammatory effects	Moderate	May increase the risk of bleeding

Melatonin	Antioxidant activity Anti-inflammatory effects Antiangiogenic action	Moderate	Drowsiness May enhance sedative effects of some drugs

Oleanolic acid	Antioxidant activity Inhibition of the formation of advanced glycation end products	Very low	Minimal toxicity

Alpha-lipoic acid	Antioxidant activity	Moderate	May interfere with anticancer, thyroid disease, and antidiabetic drugs

*Ginkgo biloba*	Antioxidant activity Anti-inflammatory effects	Moderate	May cause gastrointestinal issues

Abbreviations: NF-*κ*B, nuclear-kB transcription factor; PUFAs, polyunsaturated fatty acids; ROS, reactive oxygen species; RPE, retinal pigment epithelial.

## Data Availability

Data sharing is not applicable to this article as no new data were created or analyzed in this study.
